# An Obesity Paradox of Asian Body Mass Index after Cardiac Surgery: Arterial Oxygenations in Duration of Mechanic Ventilation

**DOI:** 10.1155/2013/426097

**Published:** 2013-09-16

**Authors:** Chiu-Hsia Chang, Fan-Yen Lee, Chin-Chou Wang, Ying-Ni Chen, Hsin-Chu Chen, Huei-Ling Hung, Meng-Chih Lin, Shih-Feng Liu

**Affiliations:** ^1^Department of Respiratory Therapy, Kaohsiung Chang Gung Memorial Hospital and Chang Gung University College of Medicine, Kaohsiung 833, Taiwan; ^2^Department of Cardiothoracic and Vascular Surgery, Kaohsiung Chang Gung Memorial Hospital and Chang Gung University College of Medicine, Kaohsiung 833, Taiwan; ^3^Division of Pulmonary and Critical Care Medicine, Department of Internal Medicine, Kaohsiung Chang Gung Memorial Hospital and Chang Gung University College of Medicine, Kaohsiung 833, Taiwan

## Abstract

*Background*. Numerous studies have documented an obesity paradox that overweight of Caucasian patients has better prognosis after cardiac surgery. This study is to examine Asian patients' BMI to see whether an obesity paradox exists in DMV after cardiac surgery. *Methods*. A retrospective study consisted of 428 patients after cardiac surgery from January 2006 to December 2010 in the medical center of Taiwan. The Asian BMI was divided into 3 groups: under-normal weight patients (BMI < 24; *n* = 165), overweight patients (BMI 24 to <27; *n* = 130), and obese patients (BMI ≥ 27; *n* = 133). Multivariable analysis and paired *t* were used to compare all variables. *Results*. Overweight patients were significantly associated with the shortest DMV. Under-normal weight patients had significantly better oxygenations of AaDO_2_ and P/F ratio in the DMV; however, they correlated with the longest DMV, older age, more female, lower LVSV, higher BUN, more dialysis-dependent, and poorer outcomes, namely, 1-year mortality, HAP, reintubation, tracheotomy, and LOS. *Conclusions*. Asian overweight patients after cardiac surgery have better prognosis. Under-normal weight patients have higher risk factors, longer DMV, and poorer outcomes; even though they have better arterial oxygenations, they seem to need better arterial oxygenations for successful weaning ventilator.

## 1. Introduction

Overweight patients having obesity paradox after cardiac surgery proposed that obesity plays a protective role for revascularization in recent years [1, 2]. There has been several studies suggesting that morbid obesity (≥40 kg/m^2^) is associated with prolonged ventilation, readmission to intensive care, and length of stay (LOS) > 14 days [3]. In the United States, underweight patients (BMI ≤ 19 kg/m^2^) face the greatest risk of mortality, prolonged ventilation, renal failure, and reoperation for bleeding [4]. Moreover, there is a more than 3-fold increase in ventilation >14 days in coronary artery bypass graft (CABG) patients who are underweight (BMI < 20 kg/m^2^) or morbidly obese (BMI > 40 kg/m^2^); in contrast, moderate-severe obese patients (BMI > 30 kg/m^2^) spend slightly less time receiving ventilation [5]. It is speculated that the patients with a BMI near 30 kg/m^2^, which is in the overweight range, had minimum risk [6]. 

The World Health Organization (WHO) establishes the BMI definitions as overweight (≥25 kg/m^2^) and obese (≥30 kg/m^2^) for Caucasians, who possess larger physiques. It is found that South Asian diabetic CABG patients have significantly higher postoperative mortality and worse overall life expectancy than diabetic Caucasian patients do [7]. However, Asian individuals have smaller physiques but higher percentage of body fat, albeit with low BMI, after the WHO revised the BMI definitions for Asian populations [8]. Therefore, the Taiwan Department of Health (DOH) has defined BMI ≥ 24 kg/m^2^ as overweight and BMI ≥ 27 kg/m^2^ as obese [9]. 

Furthermore, it is unknown whether the Asian BMI of obesity paradox exists after Asian cardiac surgery patients. Our BMI study aims to explore the duration of mechanic ventilation (DMV) of the arterial oxygenation levels for prognosis after cardiac surgery patients. 

## 2. Materials and Methods

Data were collected from a medical center in Kaohsiung Chang Gung Memorial Hospital (KCGMH), Taiwan. The Institutional Review Board of Chang Gung Medicine Foundation reviewed and approved the study protocol. 

### 2.1. Patients and Definitions

In KCGMH, 428 consecutive coronary artery disease (CAD) patients were treated with surgically isolated CABG (*n* = 329, 76.9%), and combined valve or other cardiac procedures (*n* = 99, 23.1%) during 2006–2010. 

The patients were grouped according to BMI into 3 groups; the BMI is derived from dividing the weight in kilograms by the squared height in meters (kg/m^2^), and the BMI cut-off points were based on the Taiwan DOH [9]: underweight to normal-weight group <24 kg/m^2^ (*n* = 165, 38.6%), overweight group, 24 to <27 kg/m^2^ (*n* = 130, 30.4%), and obese group ≥ 27 kg/m^2^ (*n* = 133, 31.1%).

### 2.2. Data Collection and Definitions

The following details were collected from the medical chart database. The variables of operative risk factors were age; female; number of CAD or CABG diseased coronary vessels to bypass surgery; duration of cardiopulmonary bypass (CPB) and aortic cross-clamp (ACC); habits of smoker, alcohol drinker, and betel nut chewer; history of diabetes mellitus (DM), hypertension, chronic kidney disease (CKD), dialysis dependent, unstable angina, and myocardial infarction (MI); left ventricular stroke volume (LVSV) and ejection fraction (LVEF) determined with preoperative transthoracic echocardiography; and last preoperative blood urea nitrogen (BUN) and creatinine data.

The variables of oxygenations details were AaDO_2_ and P/F ratio. Besides, the variables of outcome markers were postoperative received MV period; death within 30 days or 1 year after surgery; record of new hospital-acquired pneumonia (HAP) infection; purulent material from sternal wound infection (SWI); extubation failure after reintubation or requiring tracheotomy surgery; and length of stay (LOS) in hospital.

### 2.3. The DMV and Arterial Oxygenation Levels

Successful mechanical ventilator (MV) weaning was defined according to the Bureau of National Health Insurance and the Taiwan DOH [10]. The weaning procedure data were obtained from the medical chart database. Before surgery of invasive or noninvasive ventilation were excluded calculations of the DMV. The spontaneous breathing trial (SBT) phase was discontinuation of the ventilator for the patients' extubation. The definition of successful weaning was that nonmechanically assisted at least 5 days in the SBT phase. Thus fewer 5 days in the SBT phase as the weaning failure can be accumulated as the DMV. In addition, the short DMV was also considered when patients died or critically were ill following discharge. 

Then, the arterial blood gases (ABGs) were performed by comparing arterial blood from 2 periods: the beginning of mechanically assisted and the finally of successful SBT. The classic formulas were obtained from the ABGs and the FiO_2_ data from all patients. The alveolar-arterial oxygen tension gradient (PAO_2_-PaO_2_, AaDO_2_) formula predicted the degree of shunt; higher AaDO_2_ values indicate abnormal inefficiencies in oxygen exchange. Another friendlier formula is the ratio of arterial oxygen concentration to the fraction of inspired oxygen (PaO_2_/FiO_2_, P/F); a higher ratio indicates better gas exchange [11]. 

### 2.4. Statistical Methods

This was a cohort retrospective study that analyzed 428 patients. The chi-square test for crosstab data was used; categorical variables were expressed as proportions and percentages, and normally distributed continuous variables were presented as mean ± SD. The statistical significant differences among the 3 groups were determined with 1-way ANOVA and multiple comparisons. The arterial oxygenation results of the two phases were performed by using a paired *t*-test. A *P* value < 0.05 indicated statistical significance. The statistical analysis was performed with SPSS for Windows Version 10 (SPSS Inc., Chicago, Illinois, USA). 

## 3. Results

This study involved 428 patients.  Table 1 lists the comparison of the operative risk factors in the BMI groups; mean age was 64.7 ± 10.1 years (range: 36–89 years) and 97 patients (22.7%) were females. The median BMI was 24.6 kg/m^2^ (range: 16–38 kg/m^2^). In this study, the mean CAD was 2.8 ± 0.6, mean CABG was 3.5 ± 1.2, mean CPB was 262.7 ± 131.9 min, and mean ACC was 165.7 ± 89.1 min. In addition, 44.4% of smokers, 52.1% of patients with diabetes, 19.6% of patients with CKD, 54.9% of patients with unstable angina, and 43.5% of MI.

*Age* (*P* < 0.001) was a statistically significant difference in the BMI groups. The under-normal weight group was statistically significantly the oldest, followed by the overweight group and the obese group; multiple comparisons testing revealed that the under-normal weight group was statistically significantly older than the other two groups and overweight significantly older than the obese. In addition, the 3 groups differed in terms of the percentage of *female* predominance (*P* = 0.028); the multiple comparisons test revealed that the under-normal weight groups were statistically significantly more female-predominant than the overweight groups. The significant difference was in the percentage of *hypertension* (*P* < 0.001); multiple comparisons test revealed that the obese group had a higher prevalence of hypertension than the other 2 groups. 

Moreover, the difference in the *dialysis dependent* (*P* = 0.047) and the *BUN* (*P* = 0.013) was significant; multiple comparisons testing revealed that the dialysis dependent and the BUN in the under-normal weight those in group were higher than the obese group. By comparison, the *LVSV* (*P* < 0.001) and the *betel nut chewer* (*P* = 0.025) were statistically significantly different in terms of the BMI; multiple comparisons test revealed that the LVSV and the betel nut chewer of the under-normal weight group were statistically significantly lower than those in the obese group. 

**Figure 1:**
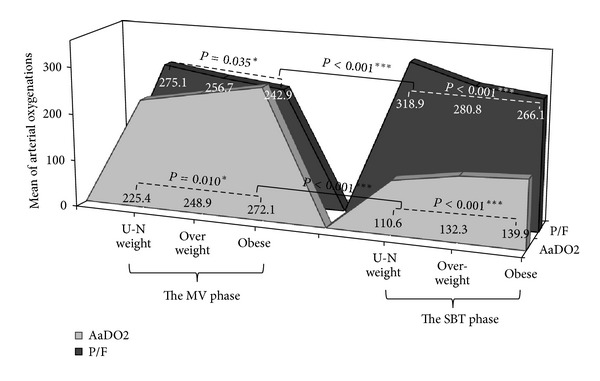
Distribution of the arterial oxygenation levels in the MV and the SBT phases of the BMI groups (see Table 2).

Table 2 lists the comparison of the arterial oxygenation levels of the MV and the SBT phases in the BMI groups. No significant differences of patient numbers were observed between two phases among the BMI groups. All patients of the SBT phase had better arterial oxygenation levels of *AaDO*_2_ (*P* < 0.001) and *P/F ratio* (*P* < 0.001) than those of the MV phase (see Figure 1). In the two phases (MV versus SBT), the *AaDO*_2_ (*P* = 0.010 versus *P* < 0.001) and *P/F ratio* (*P* = 0.035 versus *P* < 0.001) were statistically significantly different in terms of the BMI; multiple comparisons test revealed that the under-normal weight group has significantly better oxygenations than the obese group in the MV phase, and the better oxygenations than the other two groups in the SBT phase. 

**Figure 2:**
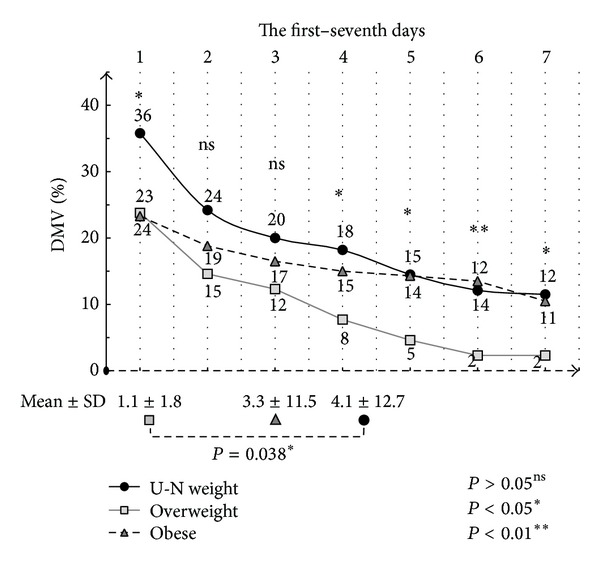
Distribution of the first–seventh days of the DMV in the BMI groups.

Table 3 describes the comparison of the outcome markers in the BMI groups. The under-normal weight patients had the longest DMV (4.1 ± 12.7 days); followed by the obese patients (3.3 ± 11.5 days), the DMV of the overweight patients was significantly lower (1.1 ± 1.8 days). In addition, there was a statistically significant difference between the *DMV* (*P* = 0.038) and BMI; the multiple comparisons test revealed that the overweight group had a statistically significantly shorter DMV than the under-normal weight group. In addition, the 3 groups' percentage of the DMV differed on the 1st, 4th, 5th, 6th, and 7th days; multiple comparisons test revealed that the overweight group had significant differences the percentage of the DMV than the other 2 groups (see Figure 2).

The difference in the percentage of *1-year mortality* was also significant (*P* = 0.004), but not 30-day mortality; multiple comparisons testing revealed that the under-normal weight group had a higher percentage of 1-year mortality than the overweight group. There was a significant difference in the percentage of *HAP* (*P* = 0.034), *reintubation* (*P* = 0.001), *tracheotomy* (*P* = 0.007), and *LOS* (*P* = 0.002); multiple comparisons testing revealed that the under-normal weight group had a higher percentage of HAP, reintubation, tracheotomy, and LOS than the other 2 groups. 

## 4. Discussion

The overweight patients have the shortest DMV. All patients of the SBT phase have better arterial oxygenation levels than the MV phase. Those who have BMI less than 24 kg/m^2^ have significantly longer DMV, higher risk factors, namely, older age, more female, lower LVSV, higher BUN, and more ratio of dialysis dependent, and poorer outcomes, namely, 1-year mortality, HAP, reintubation, tracheotomy, and LOS. Even though the under-normal weight patients have better arterial oxygenation levels, they still need more oxygenations for successful weaning ventilator. 

### 4.1. The Operative Risk Factors

Previous research had determined that in the United States, underweight patients (≤19 kg/m^2^) were at greater risk for mortality and complications after CABG surgery [4]. Similar results, operative mortality, and 5-year survival trends were similarly worse for the smallest (<24 kg/m^2^) and most severely obese patients (>34 kg/m^2^) [12]. In the present study, patients in the overweight range can be identified with minimum risk after cardiac surgery. The under-normal weight patients have more risk factors and poorer prognosis. Our findings support the premise that the BMI definition values for Asian cardiac surgery patients are similar to those of Caucasians.

Previous studies had pointed out that the small body size that reflects older age was an exaggerated drawback, and that younger age and lesser effects of CABG lead to better operative outcomes in the obese [12]. Other than this, South Asians who underwent CABG were younger, less obese, and had a higher prevalence of DM than Caucasians [7]. Moreover, being female was an independent predictor of early combined morbidity, mortality, and prolonged ICU stay [13]. 

In the present study, we agree with having the foregoing argument that the under-normal weight patients are more likely to be having smaller body size, less obese, female predominant (28.5%), older (67.7 ± 9.5 years), more percentage of DM (55.2%) and poorer outcomes. In addition, the obese patients are same as more female predominant (22.6%) and percentage of DM (52.6%), but they are more young age (61.2 ± 10.5 years).

Previous studies that assessed cardiovascular risk factors in relation to cardiac function and structure needed to account for these normal variations in the population. The normal LV differed in volume and mass between the sexes and between certain ethnic groups. When indexed by body surface area, LV mass was independent of age for both sexes [14]. The other independent risk factors were age, chronic renal failure, COPD, emergency surgery, ejection function, duration of CPB, and transfusion [15]. Moreover, cardiac dysfunction and duration of CPB had significantly interfered with the success in weaning off MV [16]. In addition, the incidence of prolonged ventilator support and operative mortality reflected preoperative medical instability, especially cardiac or respiratory insufficiency and was associated with being female, older age, and lower BMI, but not with race [5].

The present study proposed that LVEF, duration of ACC, and duration of CPB are not interfering significantly with the DMV. The LVSV in the obese patients are higher (81.2 ± 28.2 mL), but in the under-normal weight patients the LVSV are lower (69.0 ± 22.8 mL). Judging from the above, the possible explanation is that the low LVSV in the under-normal weight patients could have been due to their smaller body sizes, where the smaller diameter of their cardiac structures might affect their LV mass. In addition, the BUN provided additional information on renal function and metabolic state. The obese patients are the youngest and have the lowest BUN; by comparison, the under-normal weight patients are also reflective of the actual higher severity of illness as compared to other patients who have high BUN levels (*P* = 0.013) and higher proportion of dialysis dependent (*P* = 0.047). Consequently, the BMI is significant independent factor to determine patients' prognosis after the cardiac surgery, which is similar to the Caucasian race.

### 4.2. The Arterial Oxygenation Levels and the Outcome Markers

Previous studies suggest that hypoxemia was a common postoperative complication in cardiac surgeries, older and overweight patients, and those with left ventricular (LV) dysfunction; those who undergo prolonged cardiopulmonary bypass (CPB) face an increased risk of severe hypoxemia [17]. The hypoxemia was caused by cardiogenic and noncardiogenic pulmonary edema, pneumonia, and “hypoxemia of unknown etiology” [18]. However, the main cause of the depressed level of consciousness was prolonged sedation due to anesthetic agents and hypoxemia which was the most common cause for prolonged ventilation. That was associated with high in-hospital mortality and costs and poor 5-year survival [19]. 

In the present study, cardiac surgery patients undergoing CPB could experience increases in the body fluid content, causing an increase in lung fluid and dead space along with hypoxemia. Therefore, the ABGs provide valuable information about oxygenation, gas exchange, and lung ventilation. It is a useful method to evaluate pulmonary function and acid-base status dysfunction. Our observations also underscore the key role of oxygenation levels in the ABGs, and in that the SBT produces significantly better results in the under-normal weight patients in whom a longer DMV is indicated, providing them sufficient time to improve their physiologic or respiratory condition before being weaned off the ventilator. The overweight patients differ in terms of having lower DMV (*P* = 0.038) than the other patients.

Previous studies had reported that major abdominal surgery wounds and tissue hypoxia were common in obese patients (>30 kg/m^2^) in the perioperative period and were most pronounced during surgery. Even with supplemental oxygen, the tissue oxygen tension in obese patients was reduced to levels that were associated with a substantial increase in infection risk [20]. In the present study, it is amazing that the under-normal weight patients are significantly better associated with the oxygenation levels, but the obese patients produce poor results. It is possible that the large amount of fat in the chest wall and abdomen of the obese patients led to influences of the pulmonary mechanics, leading to longer DMV. In addition, the expanded fat tissue mass could have contributed to decreased blood flow to the cells, resulting in relative hypoperfusion with reduced tissue oxygenation and poorer oxygen parameters. 

Previous studies had found that surgical risk factors have little effect on the development of reintubation, with nasogastric tube, previous therapy with broad spectrum antibiotics, and blood transfusion being the factors most likely associated with nosocomial pneumonia acquisition [21]. Furthermore, overweight (25 to <30 kg/m^2^) and obese people (>30 kg/m^2^) had a better prognosis than those with a normal-weight (18.5 to <25 kg/m^2^) because obesity plays a protective role in revascularization for CABG patients [2]. 

In this paper, the under-normal weight group have higher 1-year mortality rates (*P* = 0.004) and have more complications considering that there is significant difference in hospital acquired pneumonia incidence (*P* = 0.034), reintubation (*P* = 0.001), tracheotomy (*P* = 0.007), and longer LOS (*P* = 0.002). However the 30-day mortality is not significantly different; however, those who are 12 of 19 patients died due to acute cardiogenic shock among the 3 groups. By comparison, Asian overweight patients with appropriate fat might have been expected to play a protective role and have less complications after cardiac surgery than the other groups. Therefore, we observe that successful early postoperative ventilator withdrawal plays a significant role in the prompt recovery of an individual's physiology. 

### 4.3. Limitations

This retrospective survey was limited because complete retrieval of patients' medical histories was only carried out at a single medical center. The 9 underweight patients (<18.5 kg/m^2^) were placed in the under-normal group, and the 6 severe obese patients (≥35 kg/m^2^) were placed in the obese group becoming an independent group. Thus, the outcome of separate analysis for these groups is unknown. However, the different groups comprised Asian cardiac surgery populations in Taiwan. As such, the patients' risks were underestimated to some extent, although we believe that this was restricted to a smaller proportion of patients. Theoretically, the predictive mortality and outcome markers of the groups would have been closer to the observed markers if such errors had been taken into consideration. 

## 5. Conclusion 

Asian BMI patients are similar to Caucasians obesity paradox views after cardiac surgery. The overweight patients have better prognosis. In contrast, the under-normal weight patients have higher risk factors and poorer outcomes, and those under-normal weight patients need better oxygenation levels and longer DMV compared with others who are overweight and obese.

## Figures and Tables

**Table 1:** Comparison of the operative risk factors in the BMI groups.

Variables	BMI (kg/m^2^)				*P* value
	U-N weight	Overweight	Obese	Total	
	<24	24 to <27	≥27		
	*n* = 165	*n* = 130	*n* = 133	*n* = 428	
Age (years)^a^	67.7 ± 9.5	64.4 ± 9.3	61.2 ± 10.5	64.7 ± 10.1	<0.001***
Female	47 (28.5)	20 (15.4)	30 (22.6)	97 (22.7)	0.028*
Isolated CABG	118 (71.5)	103 (79.2)	108 (81.2)	329 (76.9)	0.11
Number of CAD^a^	2.7 ± 0.6	2.8 ± 0.6	2.8 ± 0.5	2.8 ± 0.6	0.67
Number of CABG^a^	3.5 ± 1.3	3.6 ± 1.2	3.4 ± 1.2	3.5 ± 1.2	0.35
Duration of CPB (min)^a^	276.2 ± 148.8	265.8 ± 123.1	243.1 ± 116.6	262.7 ± 131.9	0.13
Duration of ACC (min)^a^	176.3 ± 106.8	164.4 ± 74.0	154.3 ± 78.0	165.7 ± 89.1	0.14
Smoker	74 (44.8)	54 (41.5)	62 (46.6)	190 (44.4)	0.70
Alcohol drinker	27 (16.4)	16 (12.3)	28 (21.1)	71 (16.6)	0.16
Betel nut chewer	5 (3.0)	7 (5.4)	14 (10.5)	26 (6.1)	0.025*
DM	91 (55.2)	62 (47.7)	70 (52.6)	223 (52.1)	0.44
Hypertension	98 (59.4)	78 (60.0)	107 (80.5)	283 (66.1)	<0.001***
CKD	37 (22.4)	22 (16.9)	25 (18.8)	84 (19.6)	0.48
Dialysis dependent	23 (13.9)	15 (11.5)	7 (5.3)	45 (10.5)	0.047*
Unstable angina	81 (49.1)	72 (55.4)	82 (61.7)	235 (54.9)	0.10
MI	74 (44.8)	56 (43.1)	56 (42.1)	186 (43.5)	0.89
LVSV (mL)^a^	69.0 ± 22.8	76.4 ± 22.2	81.2 ± 28.2	75.1 ± 25.0	<0.001***
LVEF (%)^a^	55.2 ± 16.8	59.2 ± 16.6	58.4 ± 16.0	57.4 ± 16.6	0.12
BUN (mg/dL)^a^	32.4 ± 27.4	26.2 ± 24.8	24.3 ± 18.5	28.0 ± 24.3	0.013*
Creatinine (mg/dL)^a^	2.3 ± 2.6	2.2 ± 2.8	1.8 ± 2.3	2.1 ± 2.6	0.20

^a^Mean ± SD; other values denote *n* (%); BMI: body mass index; U-N weight: under-normal weight; CABG: coronary artery bypass graft surgery; CAD: coronary artery disease; CPB: cardiopulmonary bypass; ACC: aortic cross-clamp; DM: diabetes mellitus; CKD: chronic kidney disease; MI: myocardial infarction; LVSV: left ventricular strove volume; LVEF: left ventricular ejection fraction; BUN: blood urea nitrogen. *P < 0.05,**P < 0.01, and ***P < 0.001.

**Table 2:** Comparison of the arterial oxygenation levels of the MV and the SBT phases in the BMI groups.

Variables	Received MV			*P* value	Received SBT			*P* value	MV versus SBT
	U-N weight	Overweight	Obese		U-N weight	Overweight	Obese		*P* value
	<24	24 to <27	≥27		<24	24 to <27	≥27		
No.	165 (100)	129 (99.2)	130 (97.7)	0.13	155 (93.9)	127 (97.7)	129 (97.0)	0.21	0.68
AaDO_2_^a^	225.4 ± 124.8	248.9 ± 135.4	272.1 ± 133.2	0.010*	110.6 ± 52.0	132.3 ± 54.6	139.9 ± 53.4	<0.001***	<0.001***
P/F ratio^a^	275.1 ± 109.0	256.7 ± 104.2	242.9 ± 104.6	0.035*	318.9 ± 97.4	280.8 ± 91.7	266.1 ± 91.9	<0.001***	<0.001***

^a^Mean ± SD; other values denote *n* (%); MV: mechanic ventilation; SBT: spontaneous breathing trail; No.: number; AaDO_2_ = (713 × FiO_2_) − (pCO_2_/0.8) − (paO_2_), (PAO_2_-PaO_2_, a higher ratio indicates hypoxemia); P/F = PaO_2_/FiO_2_ (acute lung injury (ALI) <300, acute respiratory distress syndrome (ARDS) <200). *P < 0.05,**P < 0.01, and ***P < 0.001.

**Table 3:** Comparison of the outcome markers in the BMI groups.

Variables	BMI (kg/m^2^)				*P* value
	U-N weight	Overweight	Obese	Total	
	<24	24 to <27	≥27		
	*n* = 165	*n* = 130	*n* = 133	*n* = 428	
DMV (days)^a^	4.1 ± 12.7	1.1 ± 1.8	3.3 ± 11.5	2.9 ± 10.3	0.038*
30-day mortality	12 (7.3)	4 (3.1)	3 (2.3)	19 (4.4)	0.08
1-year mortality	25 (15.2)	5 (3.8)	11 (8.3)	41 (9.6)	0.004**
HAP	29 (17.6)	11 (8.5)	13 (9.8)	53 (12.4)	0.034*
SWI	11 (6.7)	8 (6.2)	11 (8.3)	30 (7.0)	0.78
Reintubation	24 (14.5)	4 (3.1)	8 (6.0)	36 (8.4)	0.001**
Tracheotomy	8 (4.8)	0	1 (0.8)	9 (2.1)	0.007**
LOS (days)^a^	34.0 ± 36.9	22.5 ± 13.7	25.6 ± 27.0	27.9 ± 28.8	0.002**

^a^Mean ± SD; other values denote *n* (%); DMV: duration of mechanic ventilation; HAP: hospital-acquired pneumonia; SWI: sternal wound infection; LOS: length of stay. *P < 0.05, **P < 0.01, and ***P < 0.001.
